# Digitally Enhanced Nursing Discharge Teaching Reduces Postinjury Symptoms in Older Adults With Limb Fractures: A Nonrandomized Controlled Trial

**DOI:** 10.1155/jonm/9712484

**Published:** 2026-11-06

**Authors:** Santo Imanuel Tonapa, Wen-Li Hou, Bih-O Lee

**Affiliations:** ^1^ College of Nursing Kaohsiung Medical University Kaohsiung Taiwan kmu.edu.tw; ^2^ School of Nursing Galuh University Ciamis Indonesia; ^3^ Department of Medical Research Kaohsiung Medical University Hospital Kaohsiung Taiwan kmuh.org.tw; ^4^ Department of Nursing Chung Hwa University of Medical Technology Tainan Taiwan hwai.edu.tw

**Keywords:** aged, depression, digital technology, fracture, hospitals, patient discharge

## Abstract

**Background:**

Persistent postinjury symptoms such as pain, depressive symptoms, and reduced quality of life can impede rehabilitation and delay recovery in older adults with limb fractures. Digitally enhanced, nurse‐led discharge teaching presents a promising approach to address these challenges, yet its effectiveness in geriatric limb trauma remains underexplored. This study evaluated the effects of a digitally enhanced nursing discharge teaching program on pain intensity, depressive symptoms, and quality of life in older adults with limb fractures.

**Methods:**

A nonrandomized, single‐blind, parallel‐group controlled trial enrolled 90 older adults with limb fractures at a tertiary center from January 2023 to February 2024; 76 completed all assessments. The experimental group received nurse‐led discharge teaching using a teach‐back method and an interactive digital book, delivered in person three days before discharge and reinforced through telephone follow‐up over two weeks. The control group received standard discharge care. Quality of life was the primary outcome; depressive symptoms and pain intensity were secondary outcomes. Outcomes were measured at baseline, two weeks, and six weeks postdischarge. Generalized estimating equations were used to assess group‐by‐time interaction effects.

**Results:**

Of 76 completers (mean age: 69.25 ± 3.76 years; 81.6% female), attrition was nondifferential (seven per group, 15.6%). Compared to the control group at six weeks, the experimental group showed greater reductions in pain intensity (*B* = −1.95, 95% CI: [‐2.90, −0.99], *p* < 0.001) and depressive symptoms (*B* = −1.05, 95% CI: [‐1.68, −0.42], *p* = 0.001), and greater improvement in quality of life (*B* = 12.50, 95% CI: [4.34, 20.66], *p* = 0.003). No significant between‐group differences were found at the two‐week follow‐up.

**Conclusion:**

Digitally enhanced, nurse‐led discharge teaching improved pain, depressive symptoms, and quality of life in older adults with limb fractures over a six‐week period. Although short‐term effects were limited, the intervention shows promise in enhancing recovery outcomes. Additional refinement and long‐term evaluation are required before it can be implemented more widely.

**Implications for Nursing Management:**

This study highlights the value of integrating digitally enhanced nursing discharge teaching into transitional care planning, which may improve post‐injury outcomes and support recovery management in older adult patients.

**Trial Registration:** ClinicalTrials.gov identifier: NCT05176054

## 1. Introduction

Limb fracture is a leading cause of morbidity, functional decline, and mortality in older adults, with incidence rising as global populations age. By 2050, the global population aged 65 and older is projected to reach 1.5 billion, up from 703 million in 2019, with the sharpest growth in low‐ and middle‐income countries [1]. Healthcare systems in those settings will manage proportionally more older adults with traumatic injuries [2]. In Taiwan, older adults discharged after limb fracture face a particularly high risk of unplanned readmissions within 30 days, driven by inadequate management of postinjury symptoms at home [3, 4]. Each unplanned readmission extends hospital stays, increases costs, and sets back functional recovery [5].

The most common postinjury symptoms in this population are persistent pain, depressive symptoms, and reduced quality of life. Pain following limb fracture frequently persists well beyond the acute phase and can evolve into chronic pain over time, limiting mobility and functional recovery [3, 6]. Depressive symptoms are also prevalent, with studies reporting rates of approximately 23% among older adults after traumatic injury [7]. Together, these physical and psychological consequences prolong recovery, disrupt continuity of care, and contribute to long‐term functional impairment and loss of independence [3]. Prior studies conducted in Taiwan have documented that older adults with limb fractures experience significant declines in health‐related quality of life in the months following discharge, with recovery trajectories shaped by the adequacy of postdischarge symptom management [8–10].

Discharge education delivered by nurses is one of the most practical strategies for equipping patients to manage these symptoms at home. Earlier trials showed that structured nurse‐led discharge programs improve self‐management behaviors and reduce postdischarge complications in older adults after orthopedic procedures [11, 12]. Adding digital tools to discharge teaching extends these benefits. Digital platforms such as interactive e‐books and mobile applications allow patients to access personalized, multimedia‐rich educational content at any point during recovery, reinforcing learning beyond the constraints of a single in‐hospital session [13]. Features such as visual demonstrations, self‐check quizzes, and automated follow‐up reminders have been shown to increase patient engagement and adherence to postdischarge instructions in general clinical populations [14, 15]. Digital discharge teaching has also been found to reduce pain levels, alleviate depressive symptoms, and improve quality of life among older adults managing various chronic and postacute conditions [16–18].

Despite this evidence, three important gaps remain. First, existing interventions tend to address pain, depression, and quality of life in isolation rather than targeting all three within a single, integrated program [19–21]. An integrated approach matters clinically because these outcomes are interrelated: unmanaged pain amplifies depressive symptoms, and depression in turn impairs quality of life and motivation for rehabilitation [6]. Second, the use of digital platforms as the primary delivery mode for discharge education in acute trauma nursing remains limited, particularly for geriatric limb fracture patients, where cognitive and sensory factors require careful instructional design [22]. Third, evidence on digitally enhanced discharge teaching for older adults with limb fractures in Taiwan remains scarce, leaving clinicians with little population‐specific or culturally grounded guidance. This study therefore aimed to evaluate the effects of a digitally enhanced nursing discharge teaching program on pain intensity, depressive symptoms, and quality of life in older adults with limb fractures over a six‐week follow‐up period.

## 2. Methods

### 2.1. Study Setting and Design

This study was a nonrandomized, single‐blind, parallel‐group controlled trial with pre‐ and posttesting and a six‐week follow‐up. A nonrandomized design was used because the trauma and orthopedic wards share an open physical layout and nursing staff, making individual‐level randomization impractical. Randomizing within the same ward would have created an unacceptable risk of treatment contamination between participants. Participants were allocated to either the experimental or control group. Single blinding refers to the outcome assessors who remained unaware of group allocations throughout the study; participants and nursing staff were not blinded due to the nature of the intervention.

Baseline data were collected in the hospital ward (T1), with follow‐up assessments at two weeks (T2) and six weeks (T3) postdischarge. The trial took place between January 2023 and February 2024 at a medical center in Taiwan with over 1700 beds, serving as a referral hub for trauma cases within the healthcare system. The “Transparent Reporting of Evaluations with Nonrandomized Designs (TREND)” statement was used as the study’s reporting guideline (Supporting Table 1). This study was registered at ClinicalTrials.gov on 12 December 2022 (Supporting Note 1).

### 2.2. Participants and Sample Size

Convenience sampling was employed to identify eligible participants. The nurse responsible for recruitment screened injured patients admitted for over 24 h in the trauma and orthopedic wards using the hospital’s computer system. Those meeting the criteria were asked to take part and were assigned to either the control or experimental group. The inclusion criteria for limb fracture patients were as follows: (a) aged 65–74 years (the young‐old category) [23], (b) first‐time patients with limb fracture as the primary diagnosis, (c) capable of independent mobility before the injury, (d) able to communicate in Mandarin, (e) willing to participate from hospital discharge to six weeks postdischarge, (f) able to use a smartphone and have internet access, (g) a pain score greater than four on the Numeric Pain Rating Scale (NPRS), and (h) a depression score greater than four on the Geriatric Depression Scale Short Form (GDS‐SF). Patients were not included if they (a) were cognitively impaired; (b) suffered from cardiovascular, pulmonary, or cerebral insufficiency; (c) had a history of psychiatric or terminal illnesses; or (d) had visual impairments.

Power analysis for generalized estimating equations (GEEs) with two groups and three times point showed that achieving 80% power at alpha = 0.05, with a correlation of 0.50 between repeated measurements and a medium effect size (*f* = 0.16), required a minimum of 66 participants. The medium effect size was selected based on Cohen’s conventions (1988) and is consistent with effect sizes reported in interdisciplinary nursing and rehabilitation interventions for hip‐fractured older adults in Taiwan [11, 24]. A 20% attrition buffer was applied based on dropout rates observed in prior longitudinal studies of geriatric fracture patients in similar clinical settings in Taiwan [25], bringing the enrollment target to 79 participants with at least 38–39 completers per group. Because patients were admitted continuously throughout the study period, enrollment continued until the target of 76 completers was achieved, resulting in 90 initial allocations over the 14‐month recruitment period (January 2023 to February 2024).

### 2.3. Treatments

The control group received standard discharge care. The medical team provided an educational pamphlet in the ward before discharge, containing information about medications and follow‐up schedules. The experimental group received digitally enhanced discharge teaching delivered under the teach‐back method (Supporting Figure 1) by nurses with master’s degrees across two settings: (1) the hospital ward and (2) the discharge destination. The details of the intervention protocol and schedules are presented in Supporting Table 2. The first phase was conducted in the hospital ward through nurse‐led face‐to‐face education supported by an interactive digital book. Each session lasted 10–15 min and continued until discharge. The nurse interventionist applied the teach‐back method to each topic (Supporting Figure 1). In the second phase, patients accessed the digital book independently at their discharge destination, with the nurse providing reinforcement through scheduled telephone calls. These calls began on the second day of the first week and continued until the end of the second week after discharge, conducted twice weekly with each call lasting 5–10 minutes. The nurse prescribed the learning schedule and content amount for each participant.

The digital learning tool was an interactive digital book with multimedia content designed to promote engagement among older adults (Supporting Figure 2). To accommodate the visual and motor limitations common in older adults, the e‐book was designed with enlarged font sizes, high‐contrast color layouts, and simplified touch‐screen navigation. Participants could access the digital platform at any time and from any location by downloading it to a computer, reading it online, or saving it on a smartphone or tablet. The digital book included annotation features to support learning, such as notes, highlights, and bookmarks. The educational material is based on evidence from a literature review and was evaluated by an expert panel (Supporting Table 3). The content is organized into five modules: (1) a safe living guide at home, (2) wound care and pain management, (3) nutrition and dietary guidance, (4) rehabilitation instructions, and (5) psychological support. Each module includes basic and advanced questions for self‐assessment; when a participant answers incorrectly, the e‐book navigates directly to the relevant pages for review.

To maintain fidelity, the intervention was executed according to protocol. During recruitment, participants in the experimental group received a demonstration and instructions for each component of the digitally enhanced discharge teaching program. Two days after discharge, each participant received a personalized text message as a reminder for the upcoming telephone reinforcement session. Each intervention session was documented by the nurse, and the principal investigator held monthly team meetings to review the intervention process and address protocol adherence.

### 2.4. Measurements

Demographic characteristics include gender, age, marital status, education level, and employment status. Clinical data encompass activities of daily living (ADL) evaluated using the Barthel index, independence measured by the Katz index, and nutritional status evaluated by the Mini Nutritional Assessment (MNA), all sourced from medical records. In addition, injury characteristics, such as the injury severity score and mechanism of injury, were also retrieved from patient medical records.

#### 2.4.1. The NPRS

The NPRS comprises a line that is marked with numbers from 0 to 10, whereby 0 is no pain, and 10 is the worst pain imaginable. Each participant indicated the number reflecting their pain intensity at the time. The NPRS has demonstrated strong validity and reliability in older adults. Its concurrent validity is supported by a high correlation with the visual analog scale (VAS) (*r* = 0.86) and the Verbal Descriptor Scale (*r* = 0.88) [26]. The scale’s test–retest reliability was excellent over two‐week intervals among older adults. Here, the NPRS test–retest reliability was re‐evaluated and found to be acceptable, with an interclass correlation coefficient of 0.61.

#### 2.4.2. The GDS‐SF

The GDS‐SF was used for evaluating depressive symptom levels. The GDS‐SF is a screening tool for differentiating between depressed and nondepressed older adults. This scale is used in Taiwan’s clinical settings and research to assess depression in injured older adults [6]. The form is comprised of 15 items, with 10 items indicating depression when answered affirmatively, and the other five items (Questions 1, 5, 7, 11, and 13) indicating depression when answered negatively. Participants respond to each question with a simple yes (1) or no (0), resulting in a total score of 15, where higher scores reflect greater depressive symptoms. Previous review articles have indicated that this scale is a well‐established assessment tool, showing a specificity of 57%–87% and a sensitivity rate of 60%–94% [27]. In this study, the reliability of the scale was deemed excellent, with a Cronbach’s alpha of 0.85 and an interclass correlation coefficient of 0.89.

#### 2.4.3. World Health Organization Quality of Life Questionnaire (WHOQoL‐Bref)

The abbreviated “WHOQoL‐Bref” was employed to evaluate quality of life. The questionnaire was developed by the WHO through 24 international field centers [28]. The 26‐item version consists of two single‐item questions regarding overall quality of life and general health, along with 24 items organized into 4 subscales: “physical health (7 items), psychological health (6 items), social relationships (5 items), and environment (8 items)” [28]. The rating of responses is peformed using a 5‐point Likert scale, with three items (3, 4, and 26) scored in reverse. The scores for the four subscales are transformed using a formula to yield a range from 4 to 20. Each subscale score can vary between 0 and 100, and the average score across all subscale areas reflects the overall score, with a higher score showing a better quality of life [28]. The abbreviated WHOQoL is also available in Chinese. In a prior study involving injured individuals in Taiwan, the Cronbach’s alpha for the subscales varied from 0.85 to 0.90, and the overall Cronbach’s alpha for the scale was 0.88 [8]. The scale’s reliability was excellent, with Cronbach’s alpha of 0.88 and an interclass correlation coefficient of 0.66.

### 2.5. Ethical Consideration

The Institutional Review Board of Kaohsiung Medical University Chung‐Ho Memorial Hospital gave the study ethical approval on March 6, 2023, with approval number: KMUHIRB‐F(II) 20210057. The investigator evaluated the methodology, possible advantages, and risks of the study. Every participant was made aware of their right to leave the study at any point, ensuring that their treatment or services would not be adversely affected.

### 2.6. Statistical Analysis

Data analysis was conducted using SPSS Version 24.0 (IBM Corp, 2016). Descriptive statistics were used to summarize frequencies, percentages, means, and standard deviations. Baseline group differences were examined using independent *t*‐tests for continuous variables and chi‐square tests for categorical variables. GEEs with a normal distribution and identity link function were used to assess changes in outcomes across the three time points (baseline, Week 2, and Week 6). GEE was selected because it provides valid population‐averaged estimates for longitudinal repeated‐measures data and accommodates the correlated nature of within‐subject observations. An exchangeable working correlation matrix was specified, consistent with the assumptions used in the sample size calculation, and robust covariance estimators were used for inference. Interaction terms for group and time were included in all models. Baseline imbalances in ADL (Barthel index) and functional independence (Katz index) were identified in Table 1 and controlled for in an adjusted GEE model. To assess robustness, a sensitivity analysis compared unadjusted and covariate‐adjusted GEE models. Participants who did not complete all three assessments were excluded from the primary analysis. Dropout was nondifferential between groups (seven per group) and attributable to loss of contact or acute medical transfers rather than outcome‐related reasons, supporting the validity of a complete‐case approach. To examine within‐group and between‐group differences at specific time intervals, post hoc pairwise comparisons with Bonferroni correction were conducted. Statistical significance was set at *p* < 0.05.

**TABLE 1:** Demographic and clinical data of study participants.

Variables	Total (*n* = 76) mean (SD)/*n* (%)	Group		t/*χ* ^2^	*P* value
		EG (*n* = 38) mean (SD)/*n* (%)	CG (*n* = 38) mean ± SD/*n* (%)		
Gender					
Male	14 (18.4)	6 (15.8)	8 (21.1)	0.35	0.554^a^
Female	62 (81.6)	32 (84.2)	30 (78.9)		
Age, year	69.25 ± 3.76	69.05 ± 3.88	69.45 ± 3.67	−0.45	0.650^b^
Marital status					
Single/widowed	26 (34.2)	11 (28.9)	15 (39.5)	0.93	0.333^a^
Married/partnered	50 (65.8)	27 (71.1)	23 (60.5)		
Education level					
≤ 12 years	55 (72.4)	29 (76.3)	26 (68.4)	0.59	0.442^a^
> 12 years	21 (27.6)	9 (23.7)	12 (31.6)		
Caregiver					
Spouse/partner	25 (32.9)	13 (34.2)	12 (31.6)	3.40	0.335^a^
Family member	45 (59.2)	20 (52.6)	25 (65.8)		
Homecare	5 (6.6)	4 (10.5)	1 (2.6)		
Foreign care worker	1 (1.3)	1 (2.6)	0 (0.0)		
Resettlement place					
Home	58 (76.3)	33 (86.8)	25 (65.8)	5.67	0.060^a^
Institution	4 (5.3)	2 (5.3)	2 (5.3)		
Postacute care	14 (18.4)	3 (7.9)	11 (28.9)		
Injury severity score	7.87 (5.15)	6.82 (3.34)	8.92 (6.35)	−1.81	0.074^b^
1–8	42 (55.3)	24 (63.2)	18 (47.4)	4.76	0.190^a^
9–15	28 (36.8)	12 (31.6)	16 (42.1)		
16–24	3 (3.9)	2 (5.3)	1 (2.6)		
≧ 25	3 (3.9)	0 (0.0)	3 (7.9)		
Injury location					
Upper limb fracture	29 (38.2)	19 (50.0)	10 (26.3)	4.52	0.058^a^
Lower limb fracture	47 (61.8)	19 (50.0)	28 (73.7)		
Injury mechanism					
Fall	31 (40.8)	15 (39.5)	16 (42.1)	0.17	0.916^a^
Traffic accident	38 (50.0)	19 (50.0)	19 (50.0)		
Sport	7 (9.2)	4 (10.5)	3 (7.9)		
Lengths of hospital stay (days)	8.07 (6.84)	6.74 (4.01)	9.39 (8.67)	−1.71	0.092^b^
ICU admission					
No	71 (93.4)	37 (97.4)	34 (89.5)	1.93	0.165^a^
Yes	5 (6.6)	1 (2.6)	4 (10.5)		
Nutritional status	29.92 (0.27)	29.89 (0.31)	29.95 (0.23)	0.84	0.402^b^
Activity daily living	79.28 (23.43)	97.24 (8.52)	61.32 (19.44)	10.43	< 0.001**^b^**
Independence	6.67 (1.74)	7.61 (1.03)	5.74 (1.81)	5.53	< 0.001**^b^**
Pain intensity	5.29 (2.23)	5.58 (1.85)	5.00 (2.54)	1.14	0.260^b^
Depression	6.24 (1.37)	6.37 (1.30)	6.11 (1.45)	0.83	0.408^b^
Quality of life	66.45 (15.57)	65.79 (16.87)	67.10 (14.36)	−0.36	0.715^b^

*Note:* Bold values indicate statistically significant between‐group differences (*p* < 0.05).

Abbreviations: CG, control group; EG, experimental group; ICU, intensive care unit; SD, standard deviation.

^a^Chi‐square test.

^b^Independent‐sample *t*‐test.

## 3. Results

### 3.1. Demographic, Clinical Characteristics, and Outcome Variables at Baseline

During the 14‐month study period (Figure 1), 76 of the 90 initially recruited older adults completed all three assessments and were included in the final analysis. Overall attrition was 15.6% (*n* = 14), with seven dropouts per group. A comparison of baseline characteristics between the 76 completers and 14 dropouts showed no significant differences on any continuous or categorical variable (all *p* > 0.05; see Supporting Table 4). Dropout timing and reasons were also comparable across groups, supporting the assumption of nondifferential attrition.

**FIGURE 1:**
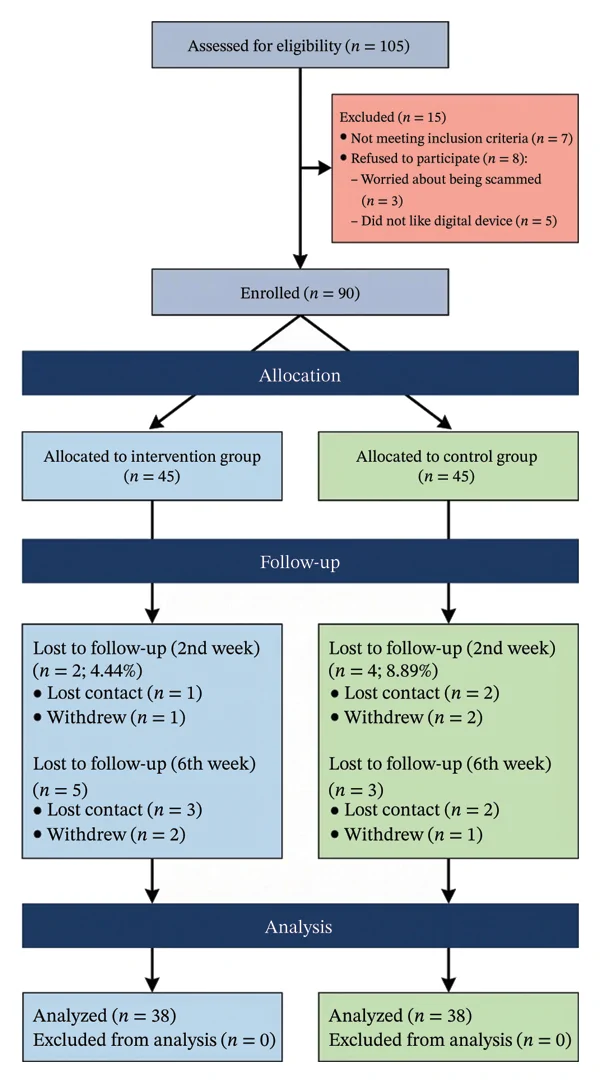
CONSORT flow diagram of research subject participation.

On average, participants from both groups were in their 60s, lived with a spouse or partner, and had an education level of 12 years or less. Most participants returned home after discharge, with family members serving as the primary caregivers. All participants sustained fracture‐related injuries, primarily involving lower limbs (61.8%), and were generally categorized as having minor trauma (55.3%). Half of these fractures resulted from traffic accidents (50%). The average length of hospital stay was 6.74 ± 4.01 days for the experimental group and 9.39 ± 8.67 days for the control group, with most participants not requiring admission to the intensive care unit (ICU).

Significant differences were noted at baseline between the two groups in terms of ADL and functional independence. Participants in the experimental group exhibited higher scores for ADL (*t* = 10.43, *p* < 0.001) and independence (*t* = 5.53, *p* < 0.001) compared to those in the control group. There were no significant differences between the two groups in terms of nutritional status, depressive symptoms, or quality of life at baseline. The between‐group difference in pain intensity at baseline did not reach significance (difference = 1.38, *p* = 0.085), with the experimental group reporting slightly higher pain scores. These baseline imbalances in ADL and independence were controlled for in the adjusted GEE model; the pain difference, while nonsignificant, is acknowledged as a potential source of residual confounding in interpreting the pain outcome. Aside from ADL and independence, the distribution of participant characteristics across both groups was homogeneous (Table 1).

### 3.2. Interaction Effect Between Group and Time on Pain Intensity, Symptoms of Depression, and Quality of Life

As indicated in Table 2, after adjusting for ADL and functional independence, the experimental group had a significantly greater decrease in pain intensity, with a difference of −1.95 (SE = 0.49, Wald *χ* ^2^ = 15.98, 95% CI: −2.90 and −0.99, *p* < 0.001) compared to the control group at six weeks. The decrease in depressive symptoms in the experimental group was greater by −1.05 (SE = 0.32, Wald *χ* ^2^ = 10.74, 95% CI: −1.68, and −0.42, *p* = 0.001) at six weeks. The improvement in quality of life for the experimental group was greater by 12.50 (SE = 4.16, Wald *χ* ^2^ = 9.02, 95% CI: 4.34 and 20.66, *p* = 0.003) at six weeks. Although the confidence interval for quality of life was wide (reflecting the high variance on this 100‐point scale; SD = 15.57), the entire interval lay above zero, confirming a significant positive effect. While the GEE interaction effect for quality of life was significant (*p* = 0.003), the post hoc between‐group comparisons at individual time points did not reach significance after Bonferroni correction (all *p* = 1.000). This reflects a difference in what these two analyses test: the GEE assesses differences in overall trajectory across all three time points simultaneously, whereas the post hoc comparisons test point‐in‐time mean differences with a conservative multiplicity adjustment across nine comparisons per outcome. No statistically significant differences were found in pain intensity (*p* = 0.157), depressive symptoms (*p* = 0.495), or quality of life (*p* = 0.857) between the two groups at the two‐week follow‐up. There were no differences between adjusted and unadjusted GEE models (Supporting Table 5).

**TABLE 2:** Covariate‐adjusted model for analysis of changes over time in pain intensity, depressive symptoms, and quality of life between the experimental and control groups.

Variables	Pain intensity			Depressive symptoms			Quality of life		
	β	95% CI	*p*	β	95% CI	*p*	β	95% CI	*p*
Intercept	6.61	[4.56, 8.66]	< 0.001	6.96	[5.47, 8.45]	< 0.001	56.84	[42.41, 71.26]	< 0.001
Treatment effect in baseline^a^									
Experimental vs. control	1.38	[0.34, 2.43]	0.009	0.55	[−0.29, 1.38]	0.199	−7.54	[−18.48, 3.41]	0.177
Time effects^b^									
6 weeks vs. baseline	−2.58	[−3.20, −1.95]	< 0.001	−0.47	[−0.71, −0.23]	< 0.001	−1.97	[−7.56, 3.61]	0.489
2 weeks vs. baseline	−2.76	[−3.48, −2.05]	< 0.001	0.05	[−0.22, 0.32]	0.705	−11.84	[−16.57, −7.11]	< 0.001
Interaction effects^c^									
Treatment^∗^time (6 weeks vs. baseline)	−1.95	[−2.90, −0.99]	**< 0.001**	−1.05	[−1.68, −0.42]	**0.001**	12.50	[4.34, 20.66]	**0.003**
Treatment^∗^time (2 weeks vs. baseline)	−0.76	[−1.82, 0.29]	0.157	−0.16	[−0.61, 0.30]	0.495	0.66	[−6.51, 7.82]	0.857
Control variable									
ADL	−0.02	[−0.03, 0.00]	0.052	0.00	[−0.02, 0.02]	0.983	0.18	[−0.09, 0.45]	0.193
Independence	−0.09	[−0.31, 0.12]	0.400	−0.15	[−0.30, 0.01]	0.060	−0.14	[−2.35, 2.07]	0.902

*Note:* Bold values indicate statistically significant group‐by‐time interaction effects (*p* < 0.05).

Abbreviations: ADL, activity of daily living; CI, confidence interval.

^a^Reference: control group.

^b^Reference: baseline measurement.

^c^Reference: control^∗^baseline measurement.

Post hoc pairwise comparisons with Bonferroni correction (Supporting Table 6) further characterized the recovery pattern. Within‐group analyses showed that both groups experienced modest changes from baseline (T1) to Week 2 (T2). Between Week 2 and Week 6 (T3), the experimental group showed statistically significant improvement across all three outcomes (pain: difference = 1.00, *p* < 0.001; depressive symptoms: difference = 1.42, *p* < 0.001; quality of life: difference = −21.71, *p* < 0.001). The control group also showed significant improvement in depressive symptoms (difference = 0.53, *p* < 0.001) and quality of life (difference = −9.87, *p* = 0.010) during the same interval, but not in pain intensity (difference = −0.18, *p* = 1.000). The magnitude of improvement between Week 2 and Week 6 was consistently greater in the experimental group across all outcomes, suggesting the intervention was associated with greater recovery than standard care during this period. Figure 2 illustrates the observed changes over time in pain intensity, depressive symptoms, and quality of life between each group.

**FIGURE 2:**
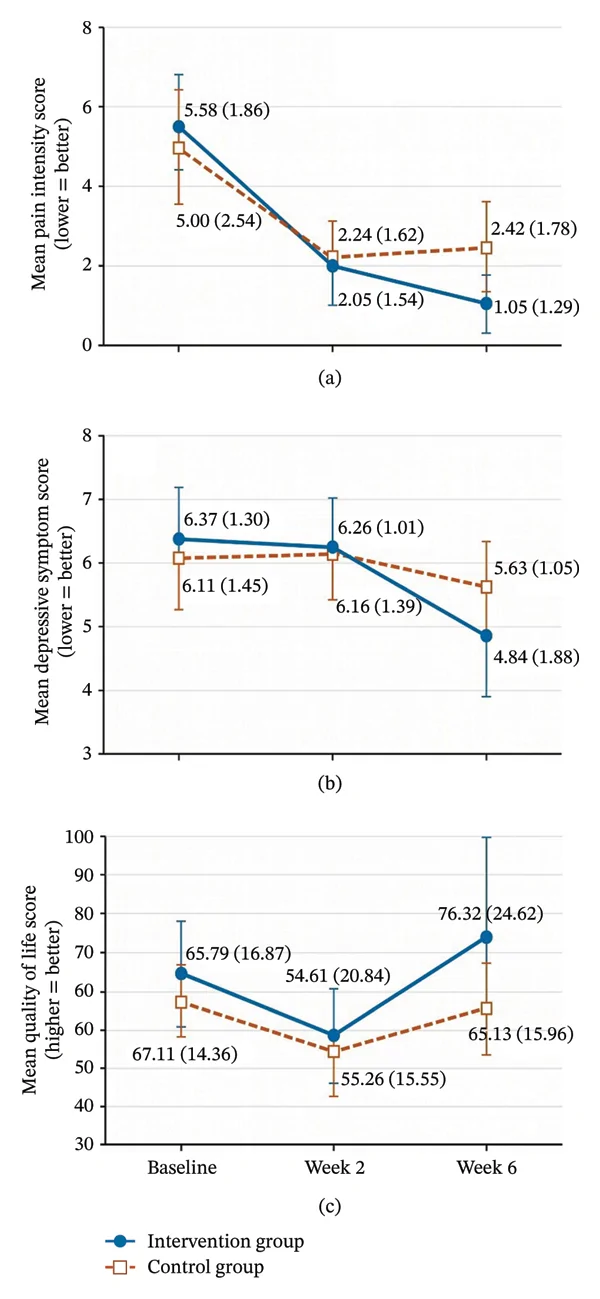
Trend of change of (a) pain, (b) depressive symptoms, and (c) quality of life.

## 4. Discussion

Older adults with limb fractures who received the digitally enhanced nursing discharge teaching program showed greater reductions in pain intensity and depressive symptoms, and greater improvements in quality of life at six weeks postdischarge, compared to those receiving standard care. No significant between‐group differences were found at the two‐week follow‐up. This delayed pattern is consistent with the broader literature on education‐based interventions, which often do not produce immediate clinical gains because knowledge retention and behavioral change take time to translate into physiological outcomes [29]. Clinically, the first two weeks after hospital discharge represent a period of high physiological stress, acute pain, and adjustment to the home environment, during which scores across both groups remained relatively similar. The greater improvement observed in the experimental group between Week 2 and Week 6 reflects the time needed for older adults to internalize the digital educational content, apply symptom management strategies, and build confidence through the structured telephone follow‐up sessions. Once this initial postdischarge vulnerability subsided, participants in the experimental group were better positioned to actively manage their recovery. Post hoc analysis confirmed that this divergence in trajectories drove the overall treatment effect, with the experimental group showing statistically significant improvement between Week 2 and Week 6 across all outcomes, while the control group improved significantly in depressive symptoms and quality of life but not in pain intensity.

The digitally enhanced discharge teaching program reduced pain intensity in injured older adults at six weeks postdischarge. This finding can be attributed to the multifaceted nature of this intervention, including its educational content, use of digital e‐books, and teach‐back method carried out both in‐hospital and at discharge destination settings via telephone reinforcement. The educational content covered pain avoidance, relief strategies, and active rehabilitation. Active rehabilitation is known to promote circulation, muscle strength, and flexibility, reducing pain and preventing complications such as joint stiffness and muscle atrophy while supporting functional restoration postinjury [30]. Digital e‐books with multimedia features may enhance engagement and promote deeper understanding and retention of pain management strategies [31, 32]. The teach‐back method gave patients repeated exposure to two‐way communication with nurses, enabling more thorough understanding of pain management knowledge and skills [33]. Together, these components likely enhanced patients’ adherence to pain management strategies, contributing to the reduction in pain scores at six weeks. Li et al. [34] and Mora‐Traverso et al. [19] reported similar reductions in pain among older adults at postintervention follow‐up, supporting the present finding. Clinicians managing older adults with limb fractures should address pain management comprehensively, combining educational content, digital tools, and structured follow‐up rather than relying on a single modality.

Older adults who received the intervention showed significantly lower depressive symptoms at six weeks, a reduction consistent with programs targeting the psychological sequelae of injury. The intervention included psychological care resources tailored to address the emotional reactions to injury in older adults [12, 19]. One module aimed to explain normal and abnormal emotional reactions after injury in older adults, equipping patients with the knowledge to recognize and normalize their experiences of sadness, lack of energy, hopelessness, or distress. It also equipped patients with strategies for coping with these emotions, contributing to the mitigation of depressive symptoms. Peng et al. [35] and Riadi et al. [36] report similar reductions, supporting the finding that interventions targeting the psychological needs of older adults can reduce depressive symptoms. These findings suggest that psychological support content should be a standard component of digital discharge teaching for injured older adults.

The digitally enhanced nursing discharge teaching intervention improved quality of life in older adults with traumatic injuries six weeks after discharge. Lin et al. [37] and Mora‐Traverso et al. [19] reported similar improvements following targeted educational programs postdischarge; the present study extends those findings by pairing an interactive e‐book with structured nurse‐led teaching and telephone follow‐up across the hospital‐to‐home transition. Unlike conventional discharge instruction, which is typically delivered once at the bedside, this approach allowed patients to revisit educational materials at their own pace throughout home recovery. Combining digital tools with structured nurse‐led teaching and telephone follow‐up may support older adults’ recovery more effectively than any single method alone. From a self‐determination theory perspective, individuals are more likely to experience intrinsic motivation and satisfaction when their needs for relatedness, competence, and autonomy are met ([38]). The program’s structured knowledge delivery, skill‐building components, and ongoing support may have addressed those psychological needs, boosting participants’ motivation for self‐care and their perceived quality of life. Clinicians managing older adults with traumatic injuries should consider integrating digital educational tools into discharge planning to support recovery outcomes.

Accessibility and engagement remain practical barriers to implementing digitally enhanced discharge teaching with older adults. Many older adults have limited experience with digital media, which may affect their willingness and ability to use digital interventions. Access barriers, including limited internet connectivity, lack of suitable devices, and physical or cognitive impairments, could hinder successful implementation [39]. Combining digital education with in‐person support may reduce these barriers. Involving caregivers or family members can further enhance engagement, while designing materials with user‐friendly interfaces, larger text, audio guidance, and simplified navigation can improve accessibility [40]. The present trial included brief in‐hospital digital literacy training before discharge, and future implementations should adopt the same approach. Given that older adults vary in technological proficiency, co‐designing the intervention to align with individual needs and abilities may improve its reach across diverse populations [41–43].

Implementation feasibility also warrants consideration. In this trial, in‐hospital teaching sessions lasted 10–15 min each, and telephone follow‐up calls averaged 5–10 minutes, conducted twice weekly over two weeks, modest enough to fit within routine nursing workflows. The long‐term benefits of improved recovery, reduced readmissions, and better adherence to care plans may outweigh initial costs, supporting the feasibility of this approach for routine clinical practice. However, the intervention was delivered by nurses with master’s‐level education. Translation to general nursing staff would require a standardized training protocol, designated follow‐up schedules, and institutional support for the associated workload. These implementation requirements should be evaluated in future pragmatic trials before recommending broader scale‐up.

### 4.1. Strengths and Limitations

This study has several strengths. The longitudinal design, with assessments at baseline, two weeks, and six weeks, allowed the recovery trajectory to be mapped over time and captured the delayed but significant improvement in outcomes that a cross‐sectional study would have missed. The intervention was implemented within an active acute trauma ward under routine clinical conditions, which strengthens the practical applicability of the findings. Attrition was low (15.6%) and evenly distributed across groups (seven dropouts per group), with baseline characteristics that did not differ between completers and dropouts, supporting the validity of the analytic sample. The use of validated, widely used outcome instruments for pain, depression, and quality of life adds to the comparability of results with the broader literature. Finally, the adjusted GEE analysis with sensitivity testing provides a conservative and statistically sound estimate of the intervention effect.

The most important limitation is the nonrandomized design, which introduces risks that must be acknowledged. Without random allocation, selection bias is possible because participants in the two groups may have differed in unmeasured characteristics such as preinjury psychosocial resilience or surgical approach; although we detected and adjusted for significant baseline differences in ADL and functional independence, unmeasured characteristics could not be controlled. The shared ward environment also introduces a risk of performance bias and treatment contamination, as nursing routines and educational delivery may have overlapped between beds in the same ward. Second, a complete‐case analytic approach was used rather than intention‐to‐treat analysis. Although this was prespecified and justified by the nondifferential nature of attrition, it means’ results reflect the intervention as delivered under complete adherence rather than effectiveness under real‐world conditions. Third, the single‐center design using convenience sampling limits generalizability. The sample also excluded patients with cognitive impairment, visual impairments, or terminal illness, which means findings may not apply to frailer or more complex patients. Fourth, the study enrolled adults aged 65 to 74 years only; the findings cannot be generalized to adults aged 75 and older, who present with greater rates of frailty, cognitive decline, and limited technological proficiency, and for whom the intervention would need adaptation before its effectiveness could be assumed. Fifth, caregiver involvement at home was not formally assessed. Given that family members may assist older adults with digital tools during recovery, the degree to which caregiver facilitation contributed to outcomes remains unknown; future studies should measure caregiver engagement as a potential moderator. Sixth, the six‐week follow‐up does not establish whether benefits persist over the longer term.

Future studies should address these limitations by employing a cluster‐randomized controlled trial design, randomizing at the ward or institutional level rather than the individual patient level, to prevent treatment contamination while preserving clinical feasibility. Independent outcome assessors, separated entirely from the care team, would further strengthen internal validity. Multisite recruitment, inclusion of adults aged 75 and older, and an extended follow‐up period of at least six months would address the remaining gaps.

## 5. Conclusion

Digitally enhanced nursing discharge teaching reduced pain intensity and depressive symptoms and improved quality of life in older adults with limb fractures over the first six weeks after discharge. This intervention produced greater recovery gains than standard discharge care, integrating interactive digital content, structured nurse‐led teaching, and telephone follow‐up. Given the nonrandomized single‐site design, these findings should be treated as preliminary evidence; controlled replication is needed before recommending broader adoption. Future research should employ a cluster‐randomized controlled trial, randomizing at the ward or institutional level to reduce treatment contamination and ensure independent outcome assessment. Multisite recruitment and a follow‐up period of at least six months would clarify whether the benefits observed at six weeks are sustained over the longer term.

## Author Contributions

Santo Imanuel Tonapa: conceptualization, methodology, investigation, data curation, formal analysis, writing–original draft, and writing–review and editing. Wen‐Li Hou: conceptualization, methodology, supervision, and writing–review and editing. Bih‐O Lee: conceptualization, funding acquisition, investigation, project administration, supervision, writing–original draft, and writing–review and editing.

## Funding

The National Science and Technology Council of Taiwan funded this research with grant number: MOST111‐2314‐B037‐023‐1.

## Disclosure

The funder had no part in the study design, conduct, or data analysis and had no authority over these activities.

## Conflicts of Interest

The authors declare no conflicts of interest.

## Supporting Information

Additional supporting information can be found online in the Supporting Information section.

## Supporting information


**Supporting Information** Supporting Note 1. Protocol adjustment notes. Supporting Table 1. TREND reporting checklist. Supporting Table 2. Intervention protocol and schedule. Supporting Table 3. Digital interactive book’s module and educational content. Supporting Table 4. Baseline comparison of demographic and clinical characteristics between completers and dropouts. Supporting Table 5. Unadjusted model for analysis of changes over time in pain intensity, depressive symptoms, and quality of life between the experimental and control groups. Supporting Table 6. Post hoc pairwise comparisons for all outcomes. Supporting Figure 1. Teach‐back method algorithm. Supporting Figure 2. Interface layout of interactive digital book.

## Data Availability

The data that support the findings of this study are available from the corresponding author upon reasonable request.
