# Supplementary material for: Digitally Enhanced Nursing Discharge Teaching Reduces Postinjury Symptoms in Older Adults With Limb Fractures: A Nonrandomized Controlled Trial
Source: J Nurs Manag. 2026 Nov 6;2026:9712484. doi: 10.1155/jonm/9712484 (PMC13743800; doi:10.1155/jonm/9712484)
Supplement: Supplementary file 1 [file JONM-2026-9712484-s001.docx]

**List of Supplementary Files**

**Supplementary Table 1.** TREND reporting checklist

**Supplementary Note 1.** Protocol adjustment notes

**Supplementary Table 2.** Intervention protocol and schedule

**Supplementary Table 3.** Digital interactive book’s module and educational content

**Supplementary Table 4.** Baseline comparison of demographic and clinical characteristics between completers and dropouts

Supplementary Table 5. Unadjusted model for analysis of changes over time in pain intensity, depressive symptoms, and quality of life between the experimental and Control groups

**Supplementary Table 6.** Post-Hoc pairwise comparisons for all outcomes

**Supplementary Figure 1.** Teach-back method algorithm

**Supplementary Figure 2.** Interface layout of interactive digital book

Supplementary Table 1. TREND reporting checklist

| **Paper Section/Topic** | **Item No.** | **Descriptor** | Reported? | |
| --- | --- | --- | --- | --- |
|  |  |  |  | Pg # |
| **TITLE and ABSTRACT** | | |  |  |
| Title and Abstract | 1 | - Information on how units were allocated to interventions | V | 1 |
|  |  | - Structured abstract recommended | V | 1 |
|  |  | - Information on target population or study sample | V | 1 |
| **INTRODUCTION** | | |  |  |
| Background | 2 | - Scientific background and explanation of rationale | V | 1-6 |
|  |  | - Theories used in designing behavioral interventions |  | N/A |
| **METHODS** | | |  |  |
| Participants | 3 | - Eligibility criteria for participants, including criteria at different levels in recruitment/sampling plan (e.g., cities, clinics, subjects) | V | 6-7 |
|  |  | - Method of recruitment (e.g., referral, self-selection), including the sampling method if a systematic sampling plan was implemented | V | 6 |
|  |  | - Recruitment setting | V | 6 |
|  |  | - Settings and locations where the data were collected | V | 6 |
| Interventions | 4 | - Details of the interventions intended for each study condition and how and when they were actually administered, specifically including: | V | 7 |
|  |  | - Content: what was given? | V | 8 and Supplementary Table 3 |
|  |  | - Delivery method: how was the content given? |  | 7 |
|  |  | - Unit of delivery: how were subjects grouped during delivery? | V | 7-8 |
|  |  | - Deliverer: who delivered the intervention? | V | 7-8, and supplementary file 2 |
|  |  | - Setting: where was the intervention delivered? | V | 7-8, and supplementary Table 2 |
|  |  | - Exposure quantity and duration: how many sessions or episodes or events were intended to be delivered? How long were they intended to last? | V | 7-8 |
|  |  | - Time span: how long was it intended to take to deliver the intervention to each unit? | V | 7-8 |
|  |  | - Activities to increase compliance or adherence (e.g., incentives) |  | N/A |
| Objectives | 5 | - Specific objectives and hypotheses | V | 6 |
| Outcomes | 6 | - Clearly defined primary and secondary outcome measures | V | 8 |
|  |  | - Methods used to collect data and any methods used to enhance the quality of measurements | V | 8-9 |
|  |  | - Information on validated instruments such as psychometric and biometric properties | V | 8-9 |
| Sample size | 7 | - How sample size was determined and, when applicable, explanation of any interim analyses and stopping rules | V | 7 |
| Assignment method | 8 | - Unit of assignment (the unit being assigned to study condition, e.g., individual, group, community) | V | 6 |
|  |  | - Method used to assign units to study conditions, including details of any restriction (e.g., blocking, stratification, minimization) |  | N/A |
|  |  | - Inclusion of aspects employed to help minimize potential bias induced due to non-randomization (e.g., matching) |  | N/A |
| Blinding (masking) | 9 | - Whether or not participants, those administering the interventions, and those assessing the outcomes were blinded to study condition assignment; if so, statement regarding how the blinding was accomplished and how it was assessed | V | 6 |
| Unit of Analysis | 10 | - Description of the smallest unit that is being analysed to assess intervention effects (e.g., individual, group, or community) | V | 11 |
|  |  | - If the unit of analysis differs from the unit of assignment, the analytical method used to account for this (e.g., adjusting the standard error estimates by the design effect or using multilevel analysis) |  | N/A |
| Statistical methods | 11 | - Statistical methods used to compare study groups for primary methods outcome(s), including complex methods for correlated data | V | 6 |
|  |  | - Statistical methods used for additional analyses, such as subgroup analyses and adjusted analysis | V | 11 |
|  |  | - Methods for imputing missing data, if used |  | N/A |
|  |  | - Statistical software or programs used | V | 11 |
| **RESULTS** | | |  |  |
| Participant flow | 12 | - Flow of participants through each stage of the study: enrollment, assignment, allocation and intervention exposure, follow-up, analysis (a diagram is strongly recommended) | V | 11 |
|  |  | - Enrollment: the numbers of participants screened for eligibility, found to be eligible or not eligible, declined to be enrolled, and enrolled in the study | V | 11-12 |
|  |  | - Assignment: the numbers of participants assigned to a study condition | V | 11-12 |
|  |  | - Allocation and intervention exposure: the number of participants assigned to each study condition and the number of participants who received each intervention | V | 11-12 |
|  |  | - Follow-up: the number of participants who completed the follow-up or did not complete the follow-up (i.e., lost to follow-up), by study condition | V | 12 and Figure |
|  |  | - Analysis: the number of participants included in or excluded from the main analysis, by study condition | X | N/A |
|  |  | - Description of protocol deviations from study as planned, along with reasons |  | N/A |
| Recruitment | 13 | - Dates defining the periods of recruitment and follow-up | V | 6 |
| Baseline data | 14 | - Baseline demographic and clinical characteristics of participants in each study condition | V | 12 and Table 1 |
|  |  | - Baseline characteristics for each study condition relevant to specific disease prevention research |  | 12-13 |
|  |  | - Baseline comparisons of those lost to follow-up and those retained, overall and by study condition | V | Supplementary Table 4 |
|  |  | - Comparison between study population at baseline and target population of interest |  | 12-13 |
| Baseline equivalence | 15 | - Data on study group equivalence at baseline and statistical methods used to control for baseline differences | V | 11 |
| Numbers analyzed | 16 | - Number of participants (denominator) included in each analysis for each study condition, particularly when the denominators change for different outcomes; statement of the results in absolute numbers when feasible | V | 7 and 12 |
|  |  | - Indication of whether the analysis strategy was “intention to treat” or, if not, description of how non-compliers were treated in the analyses | V | 12 and 20 |
| Outcomes and estimation | 17 | - For each primary and secondary outcome, a summary of results for each estimation study condition, and the estimated effect size and a confidence interval to indicate the precision | V | 13 |
|  |  | - Inclusion of null and negative findings |  | N/A |
|  |  | - Inclusion of results from testing pre-specified causal pathways through which the intervention was intended to operate, if any |  | N/A |
| Ancillary analyses | 18 | - Summary of other analyses performed, including subgroup or restricted analyses, indicating which are pre-specified or exploratory |  | N/A |
| Adverse events | 19 | - Summary of all important adverse events or unintended effects in each study condition (including summary measures, effect size estimates, and confidence intervals) |  | N/A |
| **DISCUSSION** | | |  |  |
| Interpretation | 20 | - Interpretation of the results, taking into account study hypotheses, sources of potential bias, imprecision of measures, multiplicative analyses, and other limitations or weaknesses of the study | V | 7-9 |
|  |  | - Discussion of results taking into account the mechanism by which the intervention was intended to work (causal pathways) or alternative mechanisms or explanations |  | N/A |
|  |  | - Discussion of the success of and barriers to implementing the intervention, fidelity of implementation |  | N/A |
|  |  | - Discussion of research, programmatic, or policy implications |  | N/A |
| Generalizability | 21 | - Generalizability (external validity) of the trial findings, taking into account the study population, the characteristics of the intervention, length of follow-up, incentives, compliance rates, specific sites/settings involved in the study, and other contextual issues | V | 16 |
| Overall evidence | 22 | - General interpretation of the results in the context of current evidence and current theory | V | 13 to 15 |

**Supplementary Note 1.** Protocol adjustment notes

The protocol for this research, registered on ClinicalTrials.gov (NCT05176054), was initially designed as a two-phase project: (1) development of AI-based assessment models incorporating physical balance outcomes, and (2) evaluation of a digitally enhanced nursing health service program via a randomized controlled trial.

During early implementation, the research team encountered significant technical and logistical challenges in developing and deploying the AI assessment components. After consultation with the funding agency and the Institutional Review Board (IRB), a decision was reached to formally discontinue Phase 1. This decision also required the removal of physical balance from the outcome measures, as those assessments were tied directly to the AI prediction modeling and had no independent role in Phase 2.

Concurrently, the IRB and hospital administration recommended that Phase 2 transition from a randomized to a non-randomized design. The trauma and orthopedic wards at the study site share an open physical layout and nursing staff, making individual-level randomization within the same ward impractical. Randomizing at the individual level would have introduced an unacceptable risk of treatment contamination, as nursing staff could not reliably deliver the intervention to one patient without adjacent patients becoming aware of its content.

The digitally enhanced nursing discharge teaching program evaluated in this manuscript is the originally planned Phase 2 of the project. Because Phase 1 was formally discontinued, this manuscript represents the entirety of the completed research and is not part of a larger study being published separately.

The Principal Investigator acknowledges that the ClinicalTrials.gov registry was not updated to reflect these approved protocol amendments at the time they were made. The registry has since been updated to accurately reflect the non-randomized quasi-experimental design and the finalized outcome measures of the completed study.

*Trial registration: ClinicalTrials.gov, NCT05176054. Date of original registration: 12 December 2022.*

Supplementary Table 2. Intervention protocol and schedule

| **Phase 1: In-Hospital Ward** | **Phase 2: At discharge destination** |
| --- | --- |
| **Duration:** 3 days  **Frequency:** Once daily  **Mode of Delivery:** Face-to-face teaching session with digital interactive e-book  **Activities:**   - **Day 1:**   **Introduction Session (10-15 minutes):**   - Introduction to the interactive e-book. - Identify participant’s education needs that are urgently needed. - Provide education on the safe living guide at home (Just an example, depending on the patient’s needs). - Use the teach-back method to evaluate understanding (Supplementary Figure 1 and Supplementary Figure 2). - **Day 2:**   **Teaching Session (10-15 minutes):**   - Review content from Day 1 and address any questions. - Educate on wound care and pain control (Just an example). - Reinforce the importance of timely wound dressing changes (Just an example). - Conduct a teach-back session. - **Day 3:**   **Final Teaching Session (10-15 minutes):**   - Recap on wound care and safe living at home. - Educate on nutrition, dietary instructions, and rehabilitation guidance. - Discuss psychological care and coping strategies (Just an example). - Perform a final teach-back to ensure comprehension. | **Duration:** 2 weeks  **Frequency:** Twice a week  **Mode of Delivery:** Telephone-based discharge teaching reinforcement  **Activities:**   - **Week 1:**   - **Day 1:**   **Telephone Follow-Up (5-10 minutes):**   - Prescribe participant learning schedule and dosage. - Emphasize the educational needs, focusing on safe living and wound care. - Motivate and increase confidence in using the e-book. - Assess for any problems and provide support. - **Day 4:**   **Telephone Follow-Up (5-10 minutes):**   - Review participant progress on wound care and safe living practices. - Reinforce the importance of proper nutrition and dietary habits. - Address any challenges or questions. - **Week 2:**   - **Day 8:**   **Telephone Follow-Up (5-10 minutes):**   - Evaluate the participant’s understanding and adherence to nutrition and dietary instructions. - Provide guidance on rehabilitation activities. - Offer emotional support and discuss psychological coping mechanisms. - **Day 11:**   **Telephone Follow-Up (5-10 minutes):**   - Conduct a final review of all educational content. - Assess the participant’s confidence and any ongoing needs. - Provide any additional support or information as needed. |
| **Nurse’s Role and Task Summary** | |
| - **In-Hospital:**   - Facilitate face-to-face teaching sessions.   - Utilize the digital interactive e-book for teaching.   - Identify and address individual educational needs.   - Conduct teach-back sessions to ensure comprehension. | - **At Home:**   - Perform regular telephone follow-ups.   - Reinforce educational content and support the participant’s learning.   - Address any emerging issues and provide motivational support.   - Ensure participants are confident in using the e-book and following care guidelines. |

Supplementary Figure 1. Teach-back method algorithm

Supplementary Figure 2. Interface layout of interactive digital book

Supplementary Table 3. Digital interactive book’s module and educational content

| **Module** | **Objective** | **Multimedia modality in the e-book** | **Educational content** |
| --- | --- | --- | --- |
| Safe living guide at home | To improve perceived quality of life | Images and videos | - Educate characteristics of injury and fracture site - Education on what not to do after injuries - Home environment precautions to prevent falls - Identification of high-risk medication that can cause fall-related drug use (drug review) - Assessment and education on the application of the assistive device - Prescribing an appropriate exercise - True and false test |
| Wound care and Pain control | To enhance pain control and improve perceived quality of life | Audio and images | - Wound healing process - Principle of wound care - Wound care precautions - Nature of pain - How to avoid and relieve pain - True and false test |
| Nutrition and dietary instruction | To improve perceived quality of life | Audio and images | - Post-operative dietary considerations (Do and Don't) - Progressive diet - Daily nutrient recommendation - Post-operative diet pairing test |
| Rehabilitation guidance | 1. To improve perceived quality of life and enhance pain control | Audio, images, and videos | - The rehabilitation process after injury - Precautions during the rehabilitation phase - Education for safe ambulation and mobilization after injury - Exercise recommendation after injury - True and false test |
| Psychological care | 1. To improve perceived quality of life and ameliorate depression symptoms | Audio and images | - Normal and abnormal psychological responses after a traumatic event - Cope with emotional response - Provide mental health hotlines and resources - Multiple-choice questions |

**Supplementary Table 4.** Baseline comparison of demographic and clinical characteristics between completers and dropouts

| **Variable** | **Completers (n = 76)** | **Dropouts (n = 14)** | **Test statistic** | **P value** |
| --- | --- | --- | --- | --- |
|  | *n (%) / M (SD)* | *n (%) / M (SD)* |  |  |
| **Age (years)** | 69.25 (3.76) | 69.64 (2.68) | 483.5 | .591 |
| **Gender** |  |  |  |  |
| Male | 14 (18.4%) | 4 (28.6%) |  | .467 |
| Female | 62 (81.6%) | 10 (71.4%) |  |  |
| **Marital status** |  |  |  |  |
| Single/widowed | 26 (34.2%) | 7 (50.0%) |  | .366 |
| Married/partnered | 50 (65.8%) | 7 (50.0%) |  |  |
| **Education level** |  |  |  |  |
| ≤ 12 Years | 55 (72.4%) | 12 (85.7%) |  | .505 |
| > 12 Years | 21 (27.6%) | 2 (14.3%) |  |  |
| **Caregiver** |  |  |  |  |
| Spouse/partner | 25 (32.9%) | 6 (42.9%) |  |  |
| Family member | 45 (59.2%) | 7 (50.0%) |  |  |
| Homecare | 5 (6.6%) | 1 (7.1%) |  |  |
| Foreign care worker | 1 (1.3%) | 0 (0.0%) | 0.70 | .872 |
| **Resettlement place** |  |  |  |  |
| Home | 58 (76.3%) | 13 (92.9%) |  |  |
| Institution | 4 (5.3%) | 0 (0.0%) |  |  |
| Post-acute care | 14 (18.4%) | 1 (7.1%) | 2.05 | .359 |
| **Injury severity score** | 7.87 (5.15) | 7.00 (2.72) | 560.5 | .748 |
| 1–8 | 42 (55.3%) | 9 (64.3%) |  |  |
| 9–15 | 28 (36.8%) | 5 (35.7%) |  |  |
| 16–24 | 3 (3.9%) | 0 (0.0%) |  |  |
| ≥25 | 3 (3.9%) | 0 (0.0%) | 1.28 | .734 |
| **Injury location** |  |  |  |  |
| Upper extremity | 29 (38.2%) | 8 (57.1%) |  | .240 |
| Lower extremity | 47 (61.8%) | 6 (42.9%) |  |  |
| **Injury mechanism** |  |  |  |  |
| Fall | 31 (40.8%) | 6 (42.9%) |  |  |
| Traffic accident | 38 (50.0%) | 6 (42.9%) |  |  |
| Sport | 7 (9.2%) | 2 (14.3%) | 0.44 | .802 |
| **ICU admission** |  |  |  |  |
| No | 71 (93.4%) | 12 (85.7%) |  | .298 |
| Yes | 5 (6.6%) | 2 (14.3%) |  |  |
| **Hospital stay (days)** | 8.07 (6.84) | 6.79 (3.14) | 536.5 | .964 |
| **Activity daily living** | 79.28 (23.43) | 72.86 (26.92) | 666.5 | .122 |
| **Independence (IADL)** | 6.67 (1.74) | 6.57 (1.50) | 562.0 | .717 |
| **Nutritional status** | 29.92 (0.27) | 30.00 (0.00) | 490.0 | .285 |
| **Pain intensity** | 5.29 (2.23) | 5.07 (2.23) | 537.5 | .955 |
| **Depressive symptoms** | 6.24 (1.37) | 6.21 (0.80) | 491.0 | .640 |
| **Quality of life** | 66.45 (15.57) | 62.50 (16.26) | 588.0 | .480 |

*Note.* Fisher’s exact test for 2 × 2 tables; Pearson chi-square (χ²) for larger tables; Mann-Whitney U tests (two-tailed). M = mean; SD = standard deviation. IADL = Instrumental Activities of Daily Living. ISS = Injury Severity Score. ICU = intensive care unit. PAC = post-acute care. No comparisons reached statistical significance at α = .05.

Supplementary Table 5. Unadjusted model for analysis of changes over time in pain intensity, depressive symptoms, and quality of life between the experimental and control groups

| **Variables** | **Pain intensity** | | | **Depressive symptoms** | | | **Quality of life** | | |
| --- | --- | --- | --- | --- | --- | --- | --- | --- | --- |
|  | **β** | **95% CI** | ***p*** | **β** | **95% CI** | ***p*** | **β** | **95% CI** | ***p*** |
| Intercept | 5.00 | [4.20, 5.80] | <.001 | 6.10 | [5.65, 6.56] | <.001 | 67.10 | [62.60, 71.61] | <.001 |
| Treatment effect in baseline^a^ |  |  |  |  |  |  |  |  |  |
| Experimental vs. Control | 0.57 | [-0.41, 1.56] | .250 | 0.26 | [-0.35, 0.87] | .399 | -1.32 | [-8.27, 5.63] | .711 |
| Time effects^b^ |  |  |  |  |  |  |  |  |  |
| 6 weeks vs. baseline | -2.58 | [-3.20, -1.95] | <.001 | -0.47 | [-0.71, -0.23] | <.001 | -1.97 | [-7.56, 3.61] | .489 |
| 2 weeks vs. baseline | -2.76 | [-3.48, -2.05] | <.001 | 0.05 | [-0.22, 0.32] | .705 | -11.84 | [-16.57, -7.11] | <.001 |
| Interaction effects^c^ |  |  |  |  |  |  |  |  |  |
| Treatment*time (6 weeks vs. baseline) | -1.95 | [-2.90, -0.99] | **<.001** | -1.05 | [-1.68, -0.42] | **.001** | 12.50 | [4.34, 20.66] | **.003** |
| Treatment*time (2 weeks vs. baseline) | -0.76 | [-1.82, 0.29] | .157 | -0.16 | [-0.61, 0.30] | .495 | 0.66 | [-6.51, 7.82] | .857 |
| *Note.* CI= confidence interval  ^a^Reference: control group  ^b^Reference: baseline measurement  ^c^Reference: control*baseline measurement | | | | | | | | | |

**Supplementary Table 6.** Post-Hoc pairwise comparisons for all outcomes

| **Outcome** | **Context** | **Control** | **Difference** | **Standard error** | **z** | **p value (Bonferroni)** |
| --- | --- | --- | --- | --- | --- | --- |
| **Pain intensity** | Control | T1 – T2 | 2.76 | 0.37 | 7.56 | <.001 |
|  | Control | T1 – T3 | 2.58 | 0.32 | 8.08 | <.001 |
|  | Control | T2 – T3 | −0.18 | 0.29 | −0.64 | 1.000 |
|  | Experimental | T1 – T2 | 3.53 | 0.40 | 8.89 | <.001 |
|  | Experimental | T1 – T3 | 4.53 | 0.37 | 12.30 | <.001 |
|  | Experimental | T2 – T3 | 1.00 | 0.25 | 3.96 | <.001 |
|  | Between (T1) | Exp – Con | 1.38 | 0.53 | 2.60 | .085 |
|  | Between (T2) | Exp – Con | 0.62 | 0.46 | 1.36 | 1.000 |
|  | Between (T3) | Exp – Con | −0.56 | 0.46 | −1.23 | 1.000 |
| **Depressive symptoms** | Control | T1 – T2 | −0.05 | 0.14 | −0.38 | 1.000 |
|  | Control | T1 – T3 | 0.47 | 0.12 | 3.88 | <.001 |
|  | Control | T2 – T3 | 0.53 | 0.13 | 3.96 | <.001 |
|  | Experimental | T1 – T2 | 0.11 | 0.19 | 0.57 | 1.000 |
|  | Experimental | T1 – T3 | 1.53 | 0.30 | 5.14 | <.001 |
|  | Experimental | T2 – T3 | 1.42 | 0.28 | 5.03 | <.001 |
|  | Between (T1) | Exp – Con | 0.55 | 0.43 | 1.28 | 1.000 |
|  | Between (T2) | Exp – Con | 0.39 | 0.41 | 0.94 | 1.000 |
|  | Between (T3) | Exp – Con | −0.51 | 0.49 | −1.04 | 1.000 |
| **Quality of life** | Control | T1 – T2 | 11.84 | 2.42 | 4.90 | <.001 |
|  | Control | T1 – T3 | 1.97 | 2.85 | 0.69 | 1.000 |
|  | Control | T2 – T3 | −9.87 | 3.02 | −3.27 | .010 |
|  | Experimental | T1 – T2 | 11.18 | 2.74 | 4.08 | <.001 |
|  | Experimental | T1 – T3 | −10.53 | 3.03 | −3.47 | .005 |
|  | Experimental | T2 – T3 | −21.71 | 3.50 | −6.20 | <.001 |
|  | Between (T1) | Exp – Con | −7.54 | 5.59 | −1.35 | 1.000 |
|  | Between (T2) | Exp – Con | −6.88 | 6.06 | −1.14 | 1.000 |
|  | Between (T3) | Exp – Con | 4.96 | 6.45 | 0.77 | 1.000 |

*Note.* T1 = Baseline. T2 = 2 weeks. T3 = 6 weeks. Bonferroni adjustment across 9 comparisons per outcome. Positive within-group differences indicate higher scores at the earlier time point. Positive Exp – Con differences indicate the Experimental group scored higher than control group.
